# Obesity and breast cancer outcomes in chemotherapy patients in New Zealand – a population-based cohort study

**DOI:** 10.1186/s12885-017-3971-4

**Published:** 2018-01-15

**Authors:** J. Mark Elwood, Sandar Tin Tin, Marion Kuper-Hommel, Ross Lawrenson, Ian Campbell

**Affiliations:** 1 Epidemiology and Biostatistics, School of Population Health, University of Auckland, 261 Morrin Road, Private Bag 92019, Auckland, Auckland Mail Centre 1142 New Zealand; 2 Waikato Clinical School, University of Auckland, Hamilton, New Zealand; 3 National Institute of Demographic and Economic Analysis, The University of Waikato, Hamilton, 3240 New Zealand

**Keywords:** Breast cancer, Obesity, Body-mass index, Survival, Recurrence

## Abstract

**Background:**

Obesity has been reported as an adverse prognostic factor in breast cancer, but inconsistently, and under-treatment with chemotherapy may occur. We provide the first assessment of obesity and breast cancer outcomes in a population-based, multi-ethnic cohort of New Zealand patients treated with chemotherapy.

**Methods:**

All 3536 women diagnosed with invasive breast cancer in the Waikato region of New Zealand from 2000-2014 were registered and followed until last follow-up in specialist or primary care, death or Dec 2014; median follow-up 4.1 years. For the 1049 patients receiving chemotherapy, mortality from breast cancer, other causes, and all causes, and rates of loco-regional and of distant recurrence, were assessed by body mass index (BMI), recorded after diagnosis, adjusting for other clinico-pathological and demographic factors by Cox regression.

**Results:**

BMI was known for 98% (*n*=1049); 33% were overweight (BMI 25-29.9), 21% were obese (BMI 30-34.9), and 14% were very obese (BMI 35+). There were no significant associations between obesity and survival, after adjustment for demographic and clinical factors (hazard ratios, HR, for very obese compared to BMI 21-24, for breast cancer deaths 0.96 (0.56-1.67), and for all deaths 1.03 (0.63-1.67), respectively, and only small non-significant associations for loco-regional or metastatic recurrence rates (HR 1.17 and 1.33 respectively). Subgroup analyses by age, menopausal status, ethnicity, stage, post-surgical radiotherapy, mode of diagnosis, type of surgery, and receptor status, showed no associations. No associations were seen with BMI as a continuous variable. The results in all patients irrespective of treatment but with recorded BMI data (*n*=2296) showed similar results.

**Conclusions:**

In this population, obesity assessed post-diagnosis had no effect on survival or recurrence, based on 1049 patients with chemotherapy treatment with follow-up up to 14 years.

## Background

Obesity is generally accepted as an adverse prognostic factor in breast cancer. A meta-analysis of 82 studies reported an increased risk of breast cancer mortality, hazard ratio 1.35 (95% limits 1.24-1.47) for ‘obese’ women (body mass index (BMI) 30+) compared to those with a ‘normal’ BMI (18.5 to 25) [1], seen in both pre- and post-menopausal women. This meta-analysis showed significant publication bias, suggesting that some small studies with null or inverse results have not been published. Many studies are based on incomplete or selective data: for example, one of the largest studies excluded 65% of otherwise eligible patients as they had no data on BMI recorded [2].

Several mechanisms have been suggested by which obesity could affect breast cancer prognosis; biological mechanisms influencing tumour progression; interactions with therapies; and health care-related issues affecting treatment and diagnosis.

Obesity is associated with elevated levels of serum oestrogen, produced by conversion of androgens by aromatase in adipose fat [3], and lower levels of sex hormone-binding globulin, which lowers oestrogenic activity [4]. Obesity is associated with higher levels of insulin and the adipocyte derived cytokine leptin [5] and could have effects related to markers of inflammation [6]. Effects through these mechanisms would be expected to be greater in post-menopausal women; however, in the meta-analysis noted no difference in effects by menopausal status was seen [1]. Breast cancer patients who are obese have been shown to have greater expression of proliferation genes [7], and faster growing tumours as assessed by Ki-67 [8].

These hormonal-based mechanisms suggest that anti-oestrogenic therapy might be of greater benefit to obese women. This has not been shown for tamoxifen [9], but a greater benefit from raloxifene in women with higher BMI has been suggested [10]. Obese women may have a reduced response to aromatase inhibitors [11, 12]. While the efficacy of full doses of chemotherapy does not appear to be affected by obesity [9, 13], obese women are likely to receive sub-optimal dosages of chemotherapy [14–16]. In one study in a patient population with a high prevalence of obesity, practice standards to avoid under-dosing are suggested as the reason why no effects of BMI on outcomes were seen [17].

Obese women may be disadvantaged at diagnosis; they may have larger primary tumours, more positive lymph nodes, and more advanced stage [18], and they may be less likely to be diagnosed by screening [19]. The association between BMI and breast cancer outcome may vary in women of different ethnic groups [20]. A stronger adverse effect of obesity on breast cancer survival in women of Asian ancestry has been shown in some studies [21, 22].

In this study, we assessed associations between breast cancer-specific and overall survival, and recurrence, with BMI in a large population-based cohort of women with breast cancer in New Zealand (NZ). Patients were diagnosed between January 2000 and June 2014 and followed to last follow up, death, or Dec 2016 or to death; median follow up 4.1 years. We restricted the main analysis to the 1049 patients with stage 1 to 4 breast cancer who received chemotherapy as part of their primary treatment; 98% had data on BMI collected after diagnosis but before systemic treatment. We were able to take into account age, menopausal status, ethnicity, social deprivation, comorbidity, mode of diagnosis, staging, grade, and receptor status, and primary treatment. We also assessed the outcomes in all 2296 patients, irrespective of treatment, who had known BMI data.

## Methods

### Eligible cohort

**Fig. 1:**
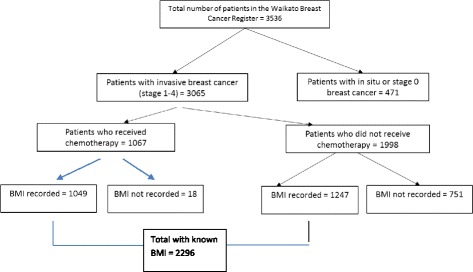
Derivation of patients for study

There were 3536 women resident in the Waikato region, New Zealand, who had breast cancer diagnosed between Jan 1, 2000 to 30 June 2014, of which 3065 had invasive disease (Fig. 1). For the main analysis, eligible women were the 1067 who had chemotherapy as part of their primary treatment. Of these 1049 (98%, all but 18) had information on height and weight before systemic treatment and were included in our main analysis. These patients were enrolled on the Waikato clinical breast cancer register and followed actively to the date of death or to last follow-up. For patients who had completed hospital-based follow up, primary care follow-up was documented. Median follow-up time was 4.1 years. The registry is linked to national mortality data and to the legally-mandated national cancer registry to ensure completeness [23], and to other hospital discharge data to assess co-morbidity. Recurrences were documented on regular hospital follow-up, or for patients discharged from regular hospital follow up, information from the primary care or private practice physician updated annually or more frequently. A secondary analysis was done on the outcomes for all 2296 women with invasive cancer who had BMI data recorded.

**Table 1:** Patient characteristics by body mass index (BMI, kg/m^2^) groups

Characteristics	Total		BMI < 21		BMI 21-24		BMI 25-29		BMI 30-34		BMI 35+		Chi-square	% Obese	Trend
	N	%	N	%	N	%	N	%	N	%	N	%	*p*-value	(BMI 30+)	*p*-value
Total	1049		81	7.7	250	23.8	349	33.3	225	21.4	144	13.7		35.2	
Adjuvant treatment															
Chemotherapy alone	338	32.2	28	8.3	78	23.1	116	34.3	77	22.8	39	11.5	0.6	34.3	
Chemotherapy and hormonal therapy	711	67.8	53	7.5	172	24.2	233	32.8	148	20.8	105	14.8		35.6	
Age															
<40	132	12.6	16	12.1	34	25.8	34	25.8	30	22.7	18	13.6	0.4	36.4	0.7
40-59	678	64.6	52	7.7	162	23.9	230	33.9	137	20.2	97	14.3		34.5	
60+	239	22.6	13	5.4	54	22.6	85	35.6	58	24.3	29	12.1		36.4	
Menopausal status															
Pre-menopause	474	45.2	44	9.3	129	27.2	150	31.6	93	19.6	58	12.2	0.06	31.9	0.13
Peri-menopause	65	6.2	0	0.0	16	24.6	22	33.8	18	27.7	9	13.8		41.5	
Post-menopause	506	48.2	37	7.3	105	20.8	176	34.8	113	22.3	75	14.8		37.2	
Missing/unknown	4	0.4	0	0.0	0	0.0	1	25	1	25.0	2	50.0		75.0	
Year of diagnosis															
2000-2002	190	18.1	16	8.4	51	26.8	66	34.7	40	21.1	17	8.9	0.5	30.0	0.2
2003-2005	213	20.3	17	8.0	52	24.4	64	30	47	22.1	33	15.5		37.6	
2006-2008	230	21.9	16	7.0	61	26.5	72	31.3	55	23.9	26	11.3		35.2	
2009-2011	218	20.8	17	7.8	40	18.3	77	35.3	48	22.0	36	16.5		38.5	
2012-2014	198	18.9	15	7.6	46	23.2	70	35.4	35	17.7	32	16.2		33.8	
Ethnicity															
European	793	75.6	69	8.7	204	25.7	283	35.7	158	19.9	79	10.0	<0.0001	29.9	
Maori	194	18.5	7	3.6	34	17.5	47	24.2	55	28.4	51	26.3		54.6	
Pacific	32	3.1	0	0.0	4	12.5	6	18.8	8	25.0	14	43.8		68.8	
Others	30	2.9	5	16.7	8	26.7	13	43.3	4	13.3	0	0.0		13.3	
Deprivation score (higher means greater deprivation)															
1-2	127	12.1	7	5.5	40	31.5	47	37	21	16.5	12	9.4	0.2	26.0	0.002
3-4	119	11.3	14	11.8	31	26.1	37	31.1	18	15.1	19	16.0		31.1	
5-6	245	23.4	18	7.3	62	25.3	84	34.3	52	21.2	29	11.8		33.1	
7-8	285	27.2	20	7.0	67	23.5	91	31.9	62	21.8	45	15.8		37.5	
9-10	272	25.9	22	8.1	49	18.0	90	33.1	72	26.5	39	14.3		40.8	
Missing/unknown	1	0.1	0	0.0	1	100.0	0	0	0	0.0	0	0.0		0.0	
Area of residence															
Urban	614	58.5	52	8.5	143	23.3	197	32.1	131	21.3	91	14.8	0.2	36.2	
Semi-urban or rural	406	38.7	29	7.1	94	23.2	144	35.5	89	21.9	50	12.3		34.2	
Missing/unknown	29	2.8	0	0.0	13	44.8	8	27.6	5	17.2	3	10.3		27.6	
Comorbidity (C3 index score)															
0	912	86.9	75	8.2	226	24.8	312	34.2	187	20.5	112	12.3	0.05	32.8	<0.001
1	75	7.1	4	5.3	13	17.3	20	26.7	21	28.0	17	22.7		50.7	
2	40	3.8	1	2.5	7	17.5	11	27.5	11	27.5	10	25.0		52.5	
3+	22	2.1	1	4.5	4	18.2	6	27.3	6	27.3	5	22.7		50.0	
Screen-detected															
Yes	303	28.9	17	5.6	57	18.8	117	38.6	72	23.8	40	13.2	0.02	37.0	
No	746	71.1	64	8.6	193	25.9	232	31.1	153	20.5	104	13.9		34.5	
Stage at diagnosis															
I	147	14	15	10.2	38	25.9	47	32	28	19.0	19	12.9	0.5	32.0	0.06
II	537	51.2	34	6.3	137	25.5	186	34.6	114	21.2	66	12.3		33.5	
III	309	29.5	25	8.1	67	21.7	97	31.4	70	22.7	50	16.2		38.8	
IV	56	5.3	7	12.5	8	14.3	19	33.9	13	23.2	9	16.1		39.3	
Grade															
I	83	7.9	7	8.4	27	32.5	27	32.5	14	16.9	8	9.6	0.6	26.5	0.7
II	512	48.8	35	6.8	120	23.4	167	32.6	113	22.1	77	15.0		37.1	
III	425	40.5	36	8.5	100	23.5	145	34.1	89	20.9	55	12.9		33.9	
Missing/unknown	29	2.8	3	10.3	3	10.3	10	34.5	9	31.0	4	13.8		44.8	
Histology															
Ductal	898	85.6	71	7.9	214	23.8	295	32.9	188	20.9	130	14.5	0.5	35.4	
Lobular	94	9	8	8.5	21	22.3	30	31.9	25	26.6	10	10.6		37.2	
Other	57	5.4	2	3.5	15	26.3	24	42.1	12	21.1	4	7.0		28.1	
ER/PR															
ER+/PR+	515	49.1	37	7.2	124	24.1	166	32.2	115	22.3	73	14.2	0.9	36.5	
ER+/PR-	216	20.6	20	9.3	49	22.7	75	34.7	38	17.6	34	15.7		33.3	
ER-/PR+	22	2.1	2	9.1	5	22.7	6	27.3	5	22.7	4	18.2		40.9	
ER-/PR-	270	25.7	20	7.4	64	23.7	92	34.1	62	23.0	32	11.9		34.8	
Missing/unknown	26	2.5	2	7.7	8	30.8	10	38.5	5	19.2	1	3.8		23.1	
HER-2															
Positive	274	26.1	20	7.3	60	21.9	90	32.8	57	20.8	47	17.2	0.7	38.0	
Equivocal	27	2.6	1	3.7	7	25.9	7	25.9	8	29.6	4	14.8		44.4	
Negative	554	52.8	47	8.5	128	23.1	189	34.1	119	21.5	71	12.8		34.3	
Missing/unknown	194	18.5	13	6.7	55	28.4	63	32.5	41	21.1	22	11.3		32.5	
Primary treatment (RT = radiotherapy)															
Breast conserving surgery with RT	451	43	29	6.4	99	22.0	152	33.7	111	24.6	60	13.3	0.1	37.9	
Breast conserving surgery, no RT	45	4.3	3	6.7	17	37.8	15	33.3	6	13.3	4	8.9		22.2	
Mastectomy with RT	379	36.1	33	8.7	93	24.5	124	32.7	69	18.2	60	15.8		34.0	
Mastectomy, no RT	130	12.4	11	8.5	35	26.9	47	36.2	24	18.5	13	10.0		28.5	
No primary surgery	44	4.2	5	11.4	6	13.6	11	25	15	34.1	7	15.9		50.0	
Total breast conserving surgery	496	47.3	32	13.1	116	59.7	167	67.0	117	37.9	64	22.2	0.3	36.5	
Total mastectomy	509	48.5	44	17.2	128	51.5	171	68.9	93	36.7	73	25.8		32.6	
Total with RT	830	79.1	62	15.1	192	46.5	276	66.4	180	42.8	120	29.1	0.02	36.1	
Total without RT	175	16.7	14	15.1	52	64.7	62	69.5	30	31.8	17	18.9		26.9	
Facility where primary treatment was undertaken															
Private	377	35.9	34	9.0	112	29.7	141	37.4	54	14.3	36	9.5	<0.0001	23.9	
Public	672	64.1	47	7.0	138	20.5	208	31.0	171	25.4	108	16.1		41.5	

Chi-sq *P* value based on table for each factor. Trend *P* value based on trend in proportion obese over ordered categories of factor

### Data

Height and weight were recorded at the first clinic visit after diagnosis and before primary treatment or after primary surgery but before systemic treatment; BMI was calculated as weight, kg/height,m^2^. Patient ethnicity was identified from the breast cancer registries or where not available from the national cancer registry or mortality data, following NZ Ministry of Health ethnicity data protocols [24]. Ethnicity was categorized into NZ European, Māori, Pacific, and Other. Socioeconomic deprivation was classified according to the New Zealand Deprivation Index 2006 [25]. This assigns small residential areas a deprivation decile on a scale of 1 to 10 based on nine socio-economic variables measured during the 2006 population census; decile1-least deprived, decile 10-most deprived. Urban/rural residential status of each woman was categorized into main urban, or other urban (independent or satellite urban) and rural, based on the New Zealand Statistics urban/rural classification system [26].

Cancer stage at diagnosis was defined according to the Tumour, Node, and Metastasis (TNM) system [27]. Invasive tumour grade was defined according to the Elston and Ellis modified Scarff-Bloom-Richardson breast cancer grading system [28]. Estrogen (ER) and progesterone (PR) receptor status was based on the results of immunohistochemistry tests and classified as positive with 1% or more receptor positive cells [29], although in years before 1999 values of 10% or more may have been used. HER-2 status was based on a Fluorescent In-Situ Hybridization (FISH) test or when this was not available, on immunohistochemistry [30]. Co-morbidity was assessed by the C3 index, using linked hospital data [31]. Menopausal status, cancer treatment variables, and local or regional recurrence were based on the reviewed clinical records. Public or private health facility was based on the place of primary treatment, usually surgery. Mortality and cause of death were based on the national cancer registry data, which incorporates clinical reviews.

### Statistical methods

Missing values except for BMI were computed using multiple imputation with ten complete datasets created by the Markov chain Monte Carlo method [32], incorporating all baseline characteristics and outcomes. Baseline data were presented as percentages, and compared across BMI groups by using chi-square and trend statistics. Cumulative incidences for specific outcomes (breast cancer specific mortality, overall mortality, death from other causes, loco-regional recurrence and metastasis) in the presence of competing risks were computed. For breast cancer specific mortality, death from other causes as the first event was considered as a competing risk. For death from other causes, breast cancer specific death as the first event was considered as a competing risk. For loco-regional recurrence and metastasis, death from any cause as the first event was considered as a competing risk. Cox proportional hazards regression modelling [33] was then performed and hazards of the specified outcomes associated with BMI were assessed. For each outcome, the proportional hazards assumption was assessed by cumulative Martingale-based residuals [34]. Hazard ratios (HRs) were adjusted for all baseline characteristics except HER-2 status (as this was assessed only after 2006): ethnicity, menopausal status, age, New Zealand Deprivation score [25], urban-rural status, mode of diagnosis (screening vs. symptomatic), year of diagnosis, stage, grade, histology, hormone receptor status (ER and PR), local treatment (surgery and radiotherapy), systemic treatment (chemotherapy, hormonal therapy and biological treatment), treatment facility (public vs. private), and C3 comorbidity index [31]. All statistical tests were two-sided and used a *p*=0.05 significance level. All analyses were performed using SAS (release 9.4, SAS Institute, Cary, North Carolina).

## Results

### Patient features and associations with BMI (patients with chemotherapy)

BMI was considered in 5 categories (Table 1). By BMI category, 81 women (7.7%) had BMI below 21 (underweight); only 8 women had BMI under 18.5. 250 (23.8%) had BMI of 21-24.9 (reference category), 349 (33.3%) had BMI from 25-29 (overweight), 225 (21.4%) had BMI 30-34.9 (obese) and 144 (13.7%) had a BMI of over 35 (very obese). Within the very obese category, 86 (8.2%) had BMI 35-39; 43 (4.1%) had BMI 40-44; 10 (1.0%) had BMI 45-49 and 5 (0.5%) BMI 50+.

**Fig. 2:**
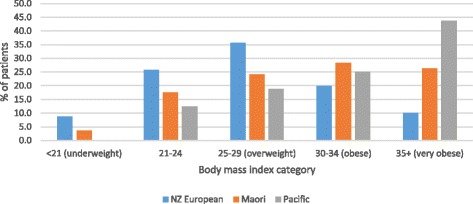
Distribution of breast cancer patients by body mass index and ethnicity (*n*=1049). Maori and Pacific distributions significantly different from NZ European (*P*< 0.001); see text

As shown in Table 1 and Fig. 2, BMI was strongly related to ethnic background, being higher in Pacific (69% over BMI 30), and Maori (55% over BMI 30) women than in NZ Europeans (30%) or other groups (mainly Asian, 13%). The distribution by BMI differs significantly between Maori and NZ Europeans and between Pacific and Europeans (both *P* values <0.001), but not between Maori and Pacific (P=0.4).

The proportion of obese (BMI 30+) patients changed little by age or menopausal status, and did not vary significantly by year of diagnosis. The proportion obese increased significantly with lower socio-economic conditions, assessed by the NZ Deprivation Code, from 26% in the least deprived to 41% in the most deprived groups. It did not vary significantly by rural or urban residence. Obesity was associated with having one or more co-morbid conditions (C3 score 1 or higher). Obesity was markedly more common in patients treated in the public health care sector (42%) than in those treated in the private sector (24%).

Obesity was more frequent in screen-detected women. By stage, 147 (14%) had stage 1 disease, 537 (51%) stage 2, 309 (30%) stage 3, and 56 (5%) stage 4. Obesity tended to be greater in women with more advanced disease, although this was not statistically significant (trend test *P*=0.06; Table 1). BMI category was not significantly related to other pathological features of grade, histology, ER, PR, or HER-2 receptor status. For primary treatment, obesity was not related to the use of breast conserving surgery compared to mastectomy. Obese patients were over-represented in women who had no primary surgery, based on small numbers. Obese patients were more likely to receive radiotherapy. Of the 1049 patients, 771 (68%) received chemotherapy and hormonal therapy, and 338 (32%) received chemotherapy alone.

### Outcomes in relation to BMI (patients with chemotherapy)

**Fig. 3:**
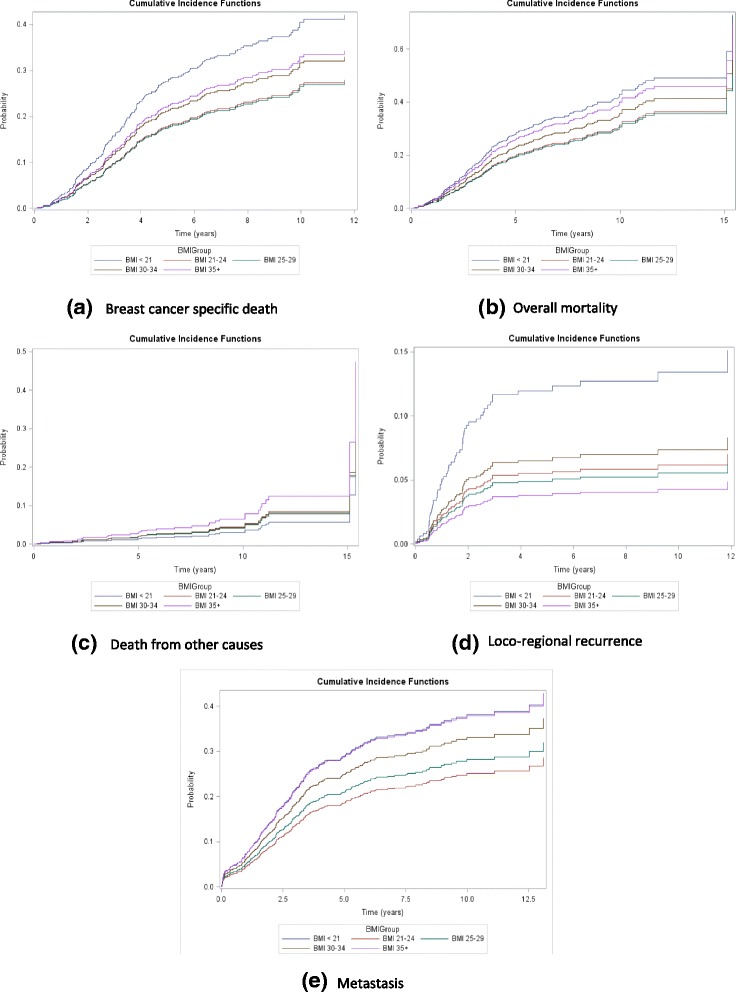
Cumulative incidence functions for breast cancer outcomes in five BMI groups: **a** breast cancer specific death, **b** overall mortality, **c** death from other causes, **d** loco-regional recurrence, **e** metastasis. See Table 2 and text for related results

Clinical outcomes were assessed for the first 10 years after diagnosis, and for the whole follow-up period up to 14 years, comparing each group to those with BMI 21-24 (Table 2 and Fig. 3). There was a trend in single-factor (unadjusted) analysis for increased hazard ratios (HR) in categories of obesity higher than the reference group of BMI 21-24 , with HR’s for very obese women being 1.28 for breast cancer deaths and 1.36 for total mortality, for the whole follow up period. Underweight women (BMI <21) also showed non-significant but increased HRs for breast cancer mortality, overall mortality, and recurrence in single factor analysis. These results are also shown as cumulative incidence curves in Fig. 3.

**Table 2:** Hazard ratios for specific breast cancer outcomes by BMI groups in patients with chemotherapy or hormonal plus chemotherapy (*N*=1049)

Outcome	Follow-up period	BMI (kg/m2)	No of events	Crude HR (95% CI)	Adjusted HR (95% CI)
Breast cancer specific death	0-10 yrs	<21 (N=81)	43	1.70 (0.99, 2.91)	1.13 (0.60, 2.14)
		21-24 (N=250)	88	1.00	1.00
		25-29 (N=349)	107	0.99 (0.66, 1.47)	0.96 (0.61, 1.51)
		30-34 (N=225)	67	1.24 (0.82, 1.88)	0.98 (0.62, 1.54)
		35+ (N=144)	49	1.31 (0.81, 2.12)	0.99 (0.57, 1.71)
	Whole study period	<21	44	1.66 (0.97, 2.84)	1.11 (0.59, 2.09)
		21-24	91	1.00	1.00
		25-29	110	0.98 (0.66, 1.46)	0.95 (0.61, 1.50)
		30-34	67	1.21 (0.80, 1.83)	0.96 (0.62, 1.50)
		35+	49	1.28 (0.79, 2.07)	0.96 (0.56, 1.67)
Overall mortality	0-10 yrs	<21	67	1.64 (0.98, 2.73)	1.12 (0.61, 2.05)
		21-24	122	1.00	1.00
		25-29	153	1.01 (0.69, 1.46)	0.92 (0.60, 1.41)
		30-34	93	1.17 (0.79, 1.73)	0.88 (0.57, 1.34)
		35+	65	1.37 (0.88, 2.16)	1.05 (0.64, 1.73)
	Whole study period	<21	70	1.49 (0.89, 2.51)	1.04 (0.57, 1.90)
		21-24	130	1.00	1.00
		25-29	163	0.97 (0.68, 1.40)	0.87 (0.57, 1.32)
		30-34	100	1.18 (0.81, 1.72)	0.89 (0.59, 1.33)
		35+	69	1.36 (0.87, 2.10)	1.03 (0.63, 1.67)
Death from other/unknown causes	0-10	<21	24	1.08 (0.22, 5.34)	1.44 (0.21, 9.97)
		21-24	34	1.00	1.00
		25-29	46	1.15 (0.41, 3.25)	1.11 (0.33, 3.72)
		30-34	26	0.74 (0.21, 2.58)	0.74 (0.21, 2.67)
		35+	16	1.64 (0.50, 5.43)	1.55 (0.34, 7.18)
	Whole study period	<21	26	0.70 (0.14, 3.45)	1.01 (0.19, 5.45)
		21-24	39	1.00	1.00
		25-29	53	0.98 (0.39, 2.43)	0.78 (0.27, 2.29)
		30-34	33	1.04 (0.40, 2.72)	1.15 (0.42, 3.11)
		35+	20	1.56 (0.54, 4.51)	1.42 (0.40, 5.01)
Loco-regional recurrence	0-5	<21 (N=81)	13	1.04 (0.22, 4.98)	1.24 (0.20, 7.64)
		21-24 (N=250)	22	1.00	1.00
		25-29 (N=349)	23	0.87 (0.32, 2.40)	1.13 (0.34, 3.73)
		30-34 (N=225)	27	0.88 (0.28, 2.77)	1.38 (0.36, 5.29)
		35+ (N=144)	11	0.60 (0.12, 2.87)	1.12 (0.16, 7.84)
	Whole study period	<21	17	1.58 (0.42, 6.02)	2.08 (0.42, 10.23)
		21-24	25	1.00	1.00
		25-29	25	1.11 (0.42, 2.93)	1.26 (0.41, 3.87)
		30-34	28	1.10 (0.37, 3.28)	1.58 (0.44, 5.63)
		35+	12	0.63 (0.13, 3.04)	1.17 (0.19, 7.21)
Distant metastasis	0-5	<21	43	1.74 (0.66, 4.54)	1.44 (0.51, 4.03)
		21-24	90	1.00	1.00
		25-29	103	1.13 (0.57, 2.24)	0.89 (0.44, 1.80)
		30-34	65	1.20 (0.56, 2.55)	1.17 (0.51, 2.67)
		35+	62	1.62 (0.72, 3.64)	1.69 (0.69, 4.11)
	Whole study period	<21	46	1.62 (0.67, 3.93)	1.32 (0.52, 3.37)
		21-24	103	1.00	1.00
		25-29	127	1.38 (0.76, 2.53)	1.05 (0.56, 1.94)
		30-34	87	1.50 (0.77, 2.90)	1.23 (0.61, 2.49)
		35+	70	1.73 (0.82, 3.63)	1.33 (0.61, 2.91)

‘Adjusted’ results from Cox regression model including BMI and ethnicity, menopausal status, age, social deprivation, urban-rural status, mode of diagnosis (screening vs. symptomatic), year of diagnosis, stage, grade, histology, hormone receptor status (ER and PR), local treatment (surgery and radiotherapy), systemic treatment (chemotherapy, hormonal therapy and biological treatment), treatment facility (public vs. private), comorbidity index

However, these associations were not significant and disappeared when other factors were taken into account, giving adjusted HRs for breast cancer-specific mortality of 0.96, 95% confidence limits 0.56 to 1.69, and for total mortality 1.03 (CI 0.63 to 1.67). There was no consistent gradient of adjusted HRs with categories of obesity. Obese women had a more elevated risk for deaths from other causes, but this was not significant after control for other factors (adjusted HR 1.55, limits 0.34 to 7.18). For loco-regional and distant recurrence, there were small increases in obese patients, but these were also not significant. For underweight women, there were no significant effects after controlling other factors. The proportional hazards assumption was met for all outcomes (Kolmogorov-type supremum test *p*-value > 0.05).

### Subgroup analyses and quantitative analysis

Further analyses of 10 year breast cancer specific mortality were carried out within subgroups of patients (Table 3), specified by age, menopausal status, ethnic group (Maori, Pacific Island, NZ European), stage (1+2, 3+4), systemic treatment (chemotherapy and hormonal therapy, chemotherapy alone), mode of detection (screening, symptomatic), receptor status (ER and PR positive, ER and PR negative, mixed), type of surgery (breast conservation, mastectomy, no surgery), and post-operative radiotherapy (yes, no). No significant and regular trends with BMI (assessed in 3 categories) were seen in any of these subgroup comparisons; in one subgroup, age 40-59, the differences were significant but there was no trend in mortality with BMI category. Results for similar for total mortality (data not shown).

**Table 3:** Subgroup analysis: breast cancer specific deaths by 10 years by BMI group and other factors

			BMI <24	BMI 25-29.9	BMI 30+	*P value*
	Total	No.	331	349	369	
Deaths 10 years, %						
All patients			19.0	15.8	19.8	*P* value
Age	<40	132	20.0	38.0	20.0	0.1
	40-59	678	18.2	10.9	19.7	0.02
	60+	239	29.2	35.4	35.4	0.9
Menopause	Pre-menopause	474	16.2	16.0	18.5	0.8
	Peri-menopause	65	6.3	13.6	11.1	0.8
	Post-menopause	506	23.9	15.9	22.3	0.2
Ethnicity*	Maori	194	26.8	21.3	24.5	0.8
	NZ European	793	18.7	15.6	17.3	0.6
Stage	1, 2	684	8.9	9.9	8.4	0.9
	3, 4	365	40.2	27.6	38.0	0.1
Detection	Screen detected	303	12.2	11.1	11.6	0.9
	Symptomatic	746	21.0	18.1	23.4	0.4
Receptors	ER+, PR+	515	13.0	12.1	14.4	0.8
	ER-, PR-	270	15.8	13.6	22.2	0.3
	+/-	238	30.1	25.0	27.7	0.7
Systemic treatment	Chemo only	338	33.0	24.1	32.8	0.2
	Chemo + hormonal	711	12.4	11.6	13.8	0.8
Surgery	Breast conserving surgery	496	14.2	11.4	9.9	0.5
	Mastectomy	509	20.4	16.4	24.1	0.2
Radiotherapy	RT	830	18.0	13.7	16.1	0.4
	no RT	175	22.4	23.6	37.5	0.09
Health sector	Public	672	22.2	16.4	20.4	0.3
	Private	377	15.1	14.9	17.8	0.8

*Notes: Ethnicity: Pacific, Asian, Other, too few for separate assessment

*P* values from chi square value for each subgroup

Survival analyses were also done using obesity as a continuous variable, excluding underweight patients, so assessing if there was any trend with increasing BMI above 21.0. For breast cancer specific mortality, the HR after controlling other factors was 0.99 (95% limits 0.96-1.02), and for overall mortality was 1.00 (0.97 to 1.03).

### Analysis of outcomes in all patients

Survival analyses were also carried out for all 2296 patients, irrespective of treatment, who had available data on BMI. No significant associations with BMI were seen, assessed in 3 categories (Table 4). The hazard ratio for the obese group (BMI 35+), compared to those with BMI under 25, for breast cancer specific death over the whole follow-up period was 1.06 (95% limits 0.80 to 1.46), and for total mortality was 0.96 (limits 0.75 to 1.21). There was an increased risk of loco-regional recurrence in the first 5 years of follow up, but this was not significant (hazard ratio 1.82, 95% limits 0.95 to 3.48). An analysis with BMI as a continuous variable also showed no association.

**Table 4:** Hazard ratios by BMI groups in all patients with known BMI (*n*=2296)

Outcome	Follow-up period	BMI (kg/m2)	Unadjusted HR (95% CI)	Adjusted HR (95% CI)
Breast cancer specific death	0-10	<25	1.00	1.00
		25-29	0.90 (0.70, 1.16)	0.98 (0.74, 1.30)
		30+	0.97 (0.76, 1.25)	1.10 (0.82, 1.46)
	Whole study period	<25	1.00	1.00
		25-29	0.90 (0.70, 1.15)	0.97 (0.73, 1.29)
		30+	0.95 (0.74, 1.22)	1.06 (0.80, 1.41)
Overall mortality	0-10	<25	1.00	1.00
		25-29	0.88 (0.71, 1.09)	1.03 (0.82, 1.30)
		30+	0.91 (0.74, 1.12)	0.96 (0.75, 1.22)
	Whole study period	<25	1.00	1.00
		25-29	0.88 (0.72, 1.08)	1.01 (0.81, 1.26)
		30+	0.93 (0.76, 1.14)	0.96 (0.76, 1.21)
Death from other/unknown causes	0-10	<25	1.00	1.00
		25-29	0.87 (0.59, 1.28)	0.97 (0.66, 1.45)
		30+	0.79 (0.53, 1.17)	0.84 (0.55, 1.29)
	Whole study period	<25	1.00	1.00
		25-29	0.89 (0.62, 1.27)	0.98 (0.68, 1.41)
		30+	0.90 (0.63, 1.29)	0.96 (0.65, 1.42)
Loco-regional recurrence	0-5	<25	1.00	1.00
		25-29	0.85 (0.42, 1.71)	0.80 (0.39, 1.67)
		30+	1.57 (0.85, 2.87)	1.82 (0.95, 3.48)
	Whole study period	<25	1.00	1.00
		25-29	0.76 (0.40, 1.44)	0.74 (0.38, 1.44)
		30+	1.35 (0.77, 2.34)	1.48 (0.81, 2.68)
Distant metastasis	0-5	<25	1.00	1.00
		25-29	0.95 (0.62, 1.46)	0.94 (0.60, 1.47)
		30+	1.15 (0.76, 1.75)	1.27 (0.81, 1.97)
	Whole study period	<25	1.00	1.00
		25-29	1.12 (0.77, 1.62)	1.08 (0.74, 1.59)
		30+	1.33 (0.92, 1.91)	1.35 (0.92, 1.98)

‘Adjusted’ results from Cox regression model including BMI and ethnicity, menopausal status, age, social deprivation, urban-rural status, mode of diagnosis (screening vs. symptomatic), year of diagnosis, stage, grade, histology, hormone receptor status (ER and PR), local treatment (surgery and radiotherapy), systemic treatment (chemotherapy, hormonal therapy and biological treatment), treatment facility (public vs. private), comorbidity index

## Discussion

This group of breast cancer patients treated with chemotherapy have a high prevalence of obesity (BMI over 30), with 35% having a BMI of 30 or greater, and 14% a BMI of 35 or greater. There was no increase in breast cancer mortality or in total mortality even in very obese women; the hazard ratio (HR) for women with a BMI of 35 or over, compared to those with BMI of 21-24, for breast cancer mortality was 0.96, with 95% confidence limits of 0.56 to 1.67, adjusted for other demographic and clinical factors; and for overall mortality, the adjusted HR was 1.03 (95% limits 0.63 to 1.67). There was no indication of a dose-response trend, either in the main analysis or in subgroup analyses, or when assessing BMI as a continuous variable throughout its range.

These results contrast with many other studies. A meta-analysis of 82 studies showed an increased risk of breast cancer mortality, HR 1.35 (95% limits 1.24-1.47) for ‘obese’ women with a BMI over 30 compared to ’normal weight’ women with a BMI between 18.5 and 25 [1]. There was a slightly greater increase in total mortality, HR 1.41 (95% limits 1.29-1.53), due mainly to a substantial, although non-significant, increase in cardiovascular mortality (HR 1.60, 95% limits 0.66-3.87). However, as noted earlier, the meta-analysis had evidence of publication bias, with Egger’s test being significant (*P*=0.03) for the studies of breast cancer mortality; the authors suggested that “small studies with inverse results are missing”.

Our results suggest that the disadvantageous prognostic effect of obesity reported elsewhere does not apply to this breast cancer population treated with chemotherapy in New Zealand. There is no clear explanation, apart from chance variation, for the contrast between these results and the results of the meta-analysis. Our data were 98% complete and based on objective clinical records after diagnosis and prior to systemic therapy. In the meta-analysis [1] differences in outcomes between obese and normal-weight women were similar for BMI assessed pre-diagnosis and within 12 months after diagnosis. However, the excess risk in underweight women was greater with post-diagnosis assessment. In the meta-analysis [1], the association of BMI with total mortality was stronger in pre-menopausal women (RR 1.75, limits 1.26-2.41) than in post-menopausal women (RR 1.34, limits 1.26-2.41), but this heterogeneity was not statistically significant. Restriction of the data to invasive, early-stage, or mammographically detected cases made little difference to the results. In our analyses, we found no associations within groups defined by menopausal status, stage, method of detection, or other clinical or demographic variables.

The main analysis presented is based on a continuous population-based clinical registry, but then restricted to patients receiving chemotherapy. For these patients information on height and weight was fully recorded, with 98% completeness. The assessment was after diagnosis and before systemic therapy was started. We are cautious about the interpretation of results for patients who did not receive chemotherapy, as there is substantial missing data, but the results were similar, not showing any association of breast cancer mortality or total mortality with BMI. However, we have shown that the missing data is not random, and is associated with survival outcomes [35]. Another New Zealand study is hard to interpret as it is based on only 27% of eligible patients because BMI data was not available on the others [36].

Some other studies have also shown no association with BMI. A study in Louisiana of 523 patients, not selected on treatment, of whom 55% were obese (BMI > 30), showed no association with overall or disease-specific survival, with a median follow-up of 49 months [17]. The authors suggested that with the high prevalence of obesity in these centres, clinicians would be more expert in dealing with obese patients and less likely, for example, to undertreat with chemotherapy. That would also apply in our population, where obesity is a prominent and familiar issue. A large randomised trial, the NSABP B-14 trial, showed no associations of breast cancer mortality with BMI [9], and under-treatment may be less likely in a trial. This trial of tamoxifen assessed 3385 women with node-negative, ER positive breast cancer, with a median follow up of 166 months. Obese women did have a higher risk of contralateral breast cancer incidence, other cancer incidence, deaths from causes other than breast cancer, and total mortality. In an analysis of 489 patients in three randomised trials of chemotherapy for metastatic breast cancer, there was no association between BMI and progression-free or overall survival [37]. Obese patients had a significantly improved progression-free survival in a study restricted to women receiving upcapped doses of chemotherapy [38]. A recent study showed more advanced staging in obese patients, but no significant effect on survival [39]. The effects of obesity on survival may only apply to certain subgroups. Thus, a study showed no overall effect on survival or recurrence, although an adverse outcome was seen in the subset of luminal A cancers [40]. In another study, obesity was associated with lower survival only in receptor positive tumours with positive lymph nodes, while it was associated with improved survival in receptor negative tumours [41]. A study of nearly 15,000 patients with pre-diagnostic BMI data showed no associations between recurrence or mortality in overweight women, and only a 10% increase in risks in obese or in severely obese women [42]. However, several more recent randomised trials of chemotherapy have shown poorer outcomes in obese women; for example [43–47].

BMI is a convenient and widely used measurement, but other assessments of body size may be relevant. A study of over 90,000 patients, assessed pre-diagnosis, showed that mortality was related to greater triceps skin fold thickness, but not to BMI [48]. In another study of post-menopausal patients, weight, but not BMI, was related to mortality [49]. Central obesity seems to have a stronger impact on African American women than general adiposity as measured by BMI [20].

BMI is related to many other lifestyle and dietary issues. In a study of 9513 breast cancer patients, mortality was related to low physical activity and to comorbidity, but not to BMI [50], although in another study BMI but not physical activity was related to increased breast cancer deaths [51]. In studies using pre-diagnosis dietary data, mortality increased regularly with intake of fat [52] in one study, and in another it increased with dietary saturated fat, and decreased with higher beta-carotene and vitamin A intakes [53].

Our study population has a substantial proportion of women of Maori and Pacific Island ethnicity (2013 census 22% and 4% respectively), the proportions in patients treated with chemotherapy being 19% and 3%; but the outcomes specific to these groups and to the majority non-Maori non-Pacific group did not show associations with BMI. A stronger effect of BMI on survival in breast cancer has been suggested for women with Asian ancestry, while effects in African Americans and Hispanics were similar to non-Hispanic whites [20], based mainly on US data. Our study population had a substantial frequency of comorbidity, including diabetes, and diabetes has been shown to be associated with a lower overall survival in breast cancer patients [54]; in our study, hazard ratios were not changed substantially by controlling for comorbidity, but the numbers were too small to assess effects specifically in those with diabetes.

## Conclusions

In summary, in a population based series of women with breast cancer in New Zealand, with 35% having a BMI of 30 or greater, no association between BMI and overall or breast cancer-specific survival, or disease-free survival, was seen in patients receiving chemotherapy, with 98% complete data on BMI; nor in all patients, irrespective of treatment, although for non-chemotherapy patients there was substantial missing data. We have no clear explanation, apart from chance variation, as to why our results differ from some other studies, but it is important to document this difference, particularly as previous meta-analyses have shown some publication bias.

## Abbreviations

BMI: Body mass index
ER: Estrogen Receptor
FISH: Fluorescent in-situ hybridization
HER-2: Human Epidermal Growth Factor Receptor 2
HR: Hazard Ratio
NZ: New Zealand
PR: Progesterone Receptor
RT: Radiotherapy
TNM: Tumour, Node, Metastasis
